# Bypassing the Blood–Brain Barrier: Direct Intracranial Drug Delivery in Epilepsies

**DOI:** 10.3390/pharmaceutics12121134

**Published:** 2020-11-24

**Authors:** Manuela Gernert, Malte Feja

**Affiliations:** 1 Department of Pharmacology, Toxicology, and Pharmacy, University of Veterinary Medicine Hannover, Bünteweg 17, D-30559 Hannover, Germany; malte.feja@tiho-hannover.de; 2 Center for Systems Neuroscience, D-30559 Hannover, Germany

**Keywords:** drug-resistant epilepsy, seizures, focal epilepsy, temporal lobe epilepsy, intracerebral drug delivery, targeted drug delivery, microinjection, microinfusion, convection-enhanced delivery, basal ganglia

## Abstract

Epilepsies are common chronic neurological diseases characterized by recurrent unprovoked seizures of central origin. The mainstay of treatment involves symptomatic suppression of seizures with systemically applied antiseizure drugs (ASDs). Systemic pharmacotherapies for epilepsies are facing two main challenges. First, adverse effects from (often life-long) systemic drug treatment are common, and second, about one-third of patients with epilepsy have seizures refractory to systemic pharmacotherapy. Especially the drug resistance in epilepsies remains an unmet clinical need despite the recent introduction of new ASDs. Apart from other hypotheses, epilepsy-induced alterations of the blood–brain barrier (BBB) are thought to prevent ASDs from entering the brain parenchyma in necessary amounts, thereby being involved in causing drug-resistant epilepsy. Although an invasive procedure, bypassing the BBB by targeted intracranial drug delivery is an attractive approach to circumvent BBB-associated drug resistance mechanisms and to lower the risk of systemic and neurologic adverse effects. Additionally, it offers the possibility of reaching higher local drug concentrations in appropriate target regions while minimizing them in other brain or peripheral areas, as well as using otherwise toxic drugs not suitable for systemic administration. In our review, we give an overview of experimental and clinical studies conducted on direct intracranial drug delivery in epilepsies. We also discuss challenges associated with intracranial pharmacotherapy for epilepsies.

## 1. Introduction

Direct drug delivery to brain structures by bypassing the blood–brain barrier (BBB) is an attractive therapeutic strategy for patients suffering from primary brain tumors and neurological disorders such as advanced Parkinson’s disease and intractable epilepsies. In our present review, we give an overview of the numerous experimental and the few clinical studies conducted on direct intracranial drug delivery in epilepsies. Epilepsies are serious chronic neurological disorders that affect more than 50 million people worldwide. Apart from the occurrence of spontaneous recurrent seizures, epilepsies can be accompanied by comorbidities such as depression, anxiety, and cognitive deficits [1]. The mainstay of epilepsy treatment is the systemic administration of antiseizure drugs (ASDs), of which there are more than 20 approved for use in humans. Apart from standard oral intake of ASDs, alternative systemic delivery approaches including intravenous (iv), subcutaneous (sc), or rectal ASD administration exist for acute seizure management such as to interrupt status epilepticus, but also for maintenance therapy during episodes of nausea and vomiting or in palliative care patients [2]. However, currently available ASDs do not cure epilepsy but instead only provide symptomatic treatment by suppressing seizures, meaning that in most cases, they must be taken life-long, imposing high demands on the safety of systemically applied ASDs. Nevertheless, patients may experience significant adverse effects, in part through the systemic ASD use [3]. Indeed, one of the factors limiting the utility of systemically administered ASDs is that peripheral organs, as well as nonepileptic brain regions, are exposed to the therapeutic drug, thereby causing systemic and/or neurological adverse effects.

Even more challenging, about 30% of epilepsy patients do not become long-term seizure-free with currently available ASDs [4], with even higher proportions for specific types such as structural epilepsies [5]. The operational definition of drug-resistant epilepsy (DRE) given by the International League Against Epilepsy (ILAE) refers to the systemic administration of two tolerated ASDs used appropriately as monotherapies or in combination at the maximum tolerated doses [6]. Apart from repeated seizure occurrence and the burden that may result from it (e.g., reduced quality of life, social stigma, loss of driving license), patients with DRE have an increased risk of injuries and premature death. Temporal lobe epilepsies (TLE) are the most common types of focal onset epilepsy and comprise the most important group of DRE in humans.

Despite the development of many new ASDs, the percentage of patients suffering from DRE has not been considerably reduced over the last decades [7,8]. In parallel to the ongoing development of new ASDs, treatment strategies alternative to systemic drug administrations have been developed and approved for clinical use, such as ketogenic diet, resective surgery and further epilepsy surgery types, vagus nerve stimulation, deep brain stimulation (DBS), and responsive neurostimulation [7,9,10,11]. However, these options offer the chance of seizure remission or reduction only for part of the patients with focal epilepsy, whose seizures continue despite ASD medications. Therefore, further alternative treatment approaches for DRE are investigated preclinically and clinically, such as neuronal transplantation, gene therapy, and the here reviewed intracranial drug delivery [12,13,14,15,16,17,18,19].

Drug-resistant epilepsies may, in fact, not necessarily be drug-resistant in all cases because the suggested mechanisms for medically refractory epilepsies include (among many others) alterations of drug uptake into the brain as well as pharmacokinetic alterations in the periphery [7,20]. We thus suggest using the term systemic drug-resistant epilepsy (sysDRE), as it seems more accurate for the strategy of direct intracranial drug delivery. Indeed, according to the drug transporter hypothesis, sysDRE is in part due to inadequate ASD passage across the BBB. Localized upregulation of multidrug efflux transporters such as P-glycoprotein in brain capillary endothelium and astrocytes of the BBB [21] in the drug-resistance epileptogenic zone [22,23] results in limited access of ASDs to the brain parenchyma, i.e., in lower extracellular concentration of ASDs at the drug target sites in the epileptogenic zone. The overexpression of these efflux transporters has been shown in the brain of DRE patients [21] as well as rodent models of refractory epilepsy [24,25]. The reduced concentration of an ASD in the seizure focus cannot be simply compensated by increasing ASD doses because of simultaneously increasing the risk of systemic and neurological adverse effects. The idea that at least with some ASDs, an antiseizure effect can be regained by inhibition of the membrane efflux transporters could be confirmed in animal models, but as yet, this is only supported by scattered clinical findings (cf., [7,20]. Furthermore, an altered expression and functionality of efflux transporters may not be restricted to the brain but could also be relevant for peripheral organs such as the intestine and liver, where this mechanism would decrease ASD plasma levels and thus the drug amount available to cross the BBB [26,27]. In addition or in a synergistic manner, an altered expression and functionality of drug-metabolizing enzymes in the periphery may contribute to persistent subtherapeutic plasma levels of ASDs described for patients with sysDRE [26,28], but not yet for animal models (cf., [20]. According to this pharmacokinetic hypothesis [27], inadequate peripheral pharmacokinetics may add to the mechanisms of sysDRE. Based on the concepts described above, circumventing the BBB and potential peripheral pharmacokinetic alterations by direct intracranial drug delivery is an attractive approach to treat sysDRE.

**Figure 1:**
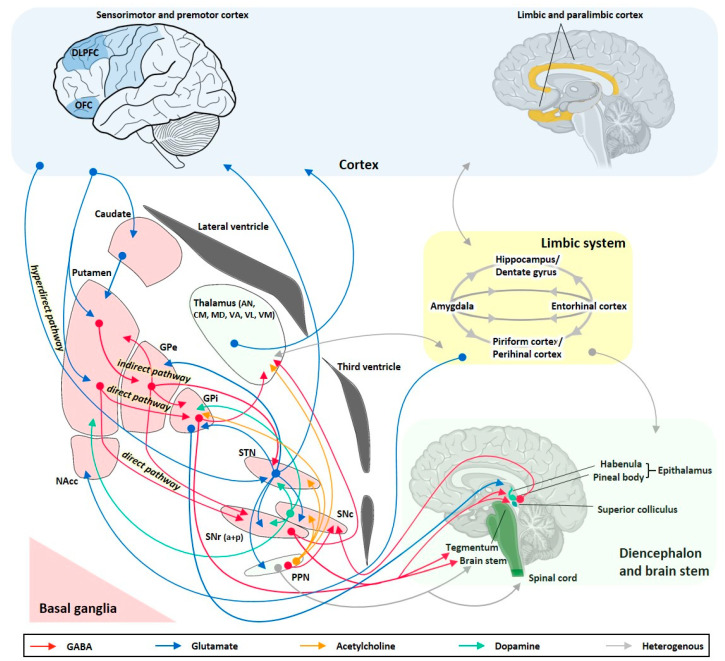
Circuitries involved in generation, propagation, and modulation of focal or focal to bilateral tonic-clonic seizures emanating from limbic/cortical structures as in temporal lobe epilepsies. For a better overview, only one hemisphere is shown, although interhemispheric epileptic networks are crucial for secondary generalization of seizures [32]. Seizure activity emanating from limbic circuitry or neocortical regions does not spread randomly through the brain but rather involves specific anatomical routes [12,33,34,35,36,37]. Targeting the seizure focus in the neocortex (highlighted in blue) or the subcortical temporal lobe network comprising hippocampus, dentate gyrus, amygdala, entorhinal, perirhinal, and piriform cortices (highlighted in yellow) is an obvious approach for intracranial drug delivery. Brain areas remote to the seizure focus play an important role in the control and propagation of different types of epilepsies/seizures. The basal ganglia (highlighted in red), especially the substantia nigra pars reticulata (SNr) and the subthalamic nucleus (STN), have been profoundly investigated in preclinical studies as a common seizure gating and control mechanism, being relatively nonselective as to the type of seizure or seizure origin they can influence [36,38,39]. Therefore, these regions are highly attractive targets for the investigation of therapeutic intracranial drug delivery approaches. Further structures, including thalamic regions and brainstem regions (highlighted in green), are also investigated in this respect. Refer to the text for more details. a+p, anterior+posterior subregions of SNr; AN, anterior thalamic nucleus; CM, centromedian thalamic nucleus; DLPFC, dorsolateral prefrontal cortex; GABA, gamma-aminobutyric acid; GPe, external globus pallidus; GPi, internal globus pallidus; MD, mediodorsal thalamus; NAcc, nucleus accumbens; OFC, orbitofrontal cortex; PPN, pedunculopontine nucleus; SNc, substantia nigra pars compacta; VA, ventral anterior nucleus; VA, ventral anterior thalamic nucleus; VL, ventral lateral thalamic nucleus; VM, ventromedial thalamic nucleus.

Furthermore, targeted intracranial administration of ASDs or other appropriate drugs may not only offer an alternative to systemic drug treatment but also to focus resection. Intracranial drug delivery approaches may be advantageous in patients in which the seizure focus is located in an eloquent brain area. The risk of irreversible functional loss, for example, of movement, speech, or sensation induced by focus resection [11], is likely reduced by targeted pharmacological suppression of pathological excitability while preserving overall cellular, white matter, and vascular structure. In addition, patients with multiple seizure foci or undefined focus localization may benefit from intracranial drug delivery into brain structures able to provide nonselective seizure control (Figure 1).

The brain networks involved in seizure generation from a limbic or neocortical focus and its propagation and modulation pathways are summarized in Figure 1. Hippocampal and extrahippocampal limbic structures such as the amygdala, entorhinal cortex, piriform cortex, and temporal neocortex are crucially involved in temporal lobe seizure generation and show multiple pathological alterations, including structural, cellular, subcellular, and molecular reorganizations associated with altered neuronal excitability [29,30,31].

The clear advantages of intracranial drug delivery over systemic drug delivery include (a) the possibility to reach higher drug concentrations in key target brain areas of the epileptic network compared to systemic administration, (b) the possibility to use otherwise toxic substances not suitable for systemic administration, (c) the reduced risk of causing systemic and/or neurological adverse effects, (d) the possibility to use substances for which the BBB is not permeable, and (e) to overcome BBB-associated drug resistance mechanisms. Disadvantages include (a) the invasive procedure necessary for intracranial drug delivery, (b) the difficulties associated with the need for long-term administration of an appropriate drug, and (c) the difficulties associated with the fact that the distribution of an appropriate drug within the brain parenchyma cannot be reliably set to the desired range. Nevertheless, intracranial pharmacotherapy is a network-specific treatment approach, which aims to specifically target the epileptic network underlying epilepsy.

**Figure 2:**
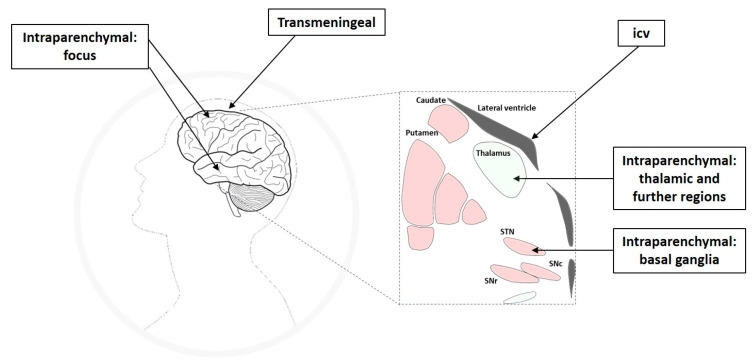
Overview of application routes for intracranial drug delivery in epilepsies resistant to systemically administered antiseizure drugs. Drugs can be delivered to the brain, aiming to target the seizure focus directly (intraparenchymal route) or indirectly (intracerebroventricular, icv, and transmeningeal route). Furthermore, drugs can be targeted directly (intraparenchymal route) to epileptic network structures remote to the seizure focus, such as basal ganglia or thalamic regions.

Intracranial drug infusion has been studied by targeting different compartments of the central nervous system (CNS), i.e., via the intraparenchymal, ventricular, or transmeningeal route. Apart from the seizure focus, seizure propagation pathways or circuits belong to the epileptic network and have been shown to offer suitable anatomical targets for therapeutic interventions (Figure 1), which will be discussed in the respective subsections of this review. The thorough definition of the optimum intracranial site for targeted drug delivery in epilepsies has been (and still is) one aspect elaborated in many of the studies reviewed here. In this respect, studies can be categorized into different approaches of intracranial drug delivery, aiming to treat sysDRE. These approaches are (a) intracerebroventricular (icv) administration to target a seizure focus in the vicinity of the ventricles, (b) transmeningeal administration to target a neocortical seizure focus, (c) intraparenchymal administration to directly target the seizure focus, and (d) intraparenchymal administration to target key seizure-modulating remote structures of the epileptic network such as basal ganglia or thalamus (Figure 2). We organized the review according to this categorization. Additionally, intracranial drug delivery studies could be subdivided concerning animal model versus clinical (human) study, used drug (ASD versus non-ASD, gamma-aminobutyric acid (GABA)ergic versus non-GABAergic), and drug delivery technique. Concerning microinfusion techniques, the differentiation between studies applying acute versus chronic administration is of relevance, because just like with systemic ASD therapy, intracranial drug therapy of epilepsies provides symptomatic seizure suppression rather than a cure.

The present review is about intracranial drug delivery as a strategy to overcome drug-resistant focal epilepsies with or without secondary generalization, because the majority of patients with DRE suffer from focal onset epilepsy. The generation of other epilepsy types such as absence epilepsies involves different cortical and subcortical brain circuitries and mechanisms [40]. In addition, ASDs effective against absence seizures often differ from ASDs effective against focal or tonic-clonic seizures. In the present review, we therefore only occasionally address absence epilepsies, although many studies showed the effectiveness of targeted drug delivery in those models as well [36,41]. Again mediated by different networks and mechanisms, we do not address studies on models of reflex epilepsies in this review, although many preclinical studies successfully investigated intracranial drug delivery in models of audiogenic seizures [42]. In our review, we will first briefly describe technical and animal model aspects of intracranial drug delivery. Next, we will review animal and human studies for the different application routes/target sites, and finally, we will discuss challenges associated with intracranial drug delivery in epilepsies.

## 2. Technical Considerations

**Figure 3:**
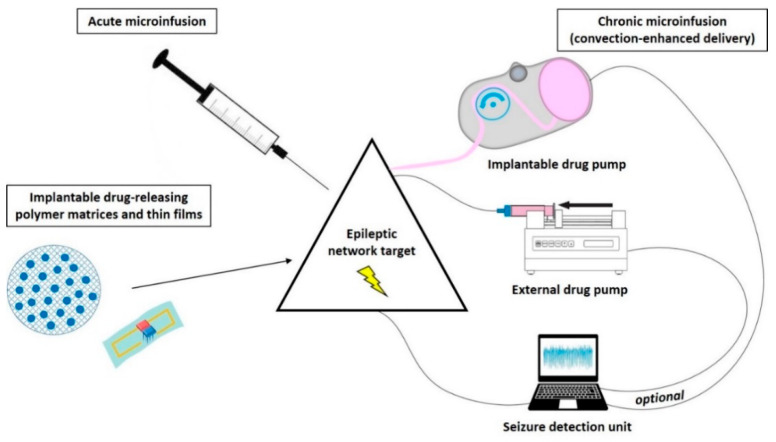
Overview showing different intracranial drug delivery techniques used to modulate epileptic brain networks. Drugs can be delivered acutely for proof-of-principle or chronically into or close to the target region of the epileptic network by controlled-release polymers (e.g., wafers, microparticles) or by an implanted catheter/cannula combined with a drug reservoir or connected to a (subcutaneously) implanted or external microinfusion pump. Depending on the technique, the spread of the drug into the brain parenchyma occurs by passive diffusion or convection-enhanced distribution. In the case of programmable microinfusion pumps, discontinuous (intermittent) drug release is achievable, thereby reducing the risk of development of pharmacological tolerance. Adding a seizure prediction/detection unit offers the possibility of responsive (closed-loop) drug delivery. For details, refer to the text.

Different technical strategies aim to achieve network-specific pharmacological seizure-control in contrast to systemic drug treatment. Concerning direct intracranial drug delivery, several techniques (Figure 3) to administer drugs locally to the brain are investigated and discussed in detail in numerous excellent reviews, e.g., [13,43,44,45,46,47,48,49,50,51,52].

Most proof-of-concept animal studies utilize acute on-site injections (bolus deposition) via stereotaxically implanted cannulas into the target region, while chronic (continuous or discontinuous) drug release is required for further development of this treatment strategy aiming to achieve long-term seizure control. An approach to prolong the duration of antiseizure effects by intracranial drug delivery is the use of drug-loaded biodegradable or nonbiodegradable polymer-based implants or bioceramics slowly releasing ASDs or other appropriate compounds, which then passively diffuse through the surrounding tissue. These drug carriers are experimentally implanted at seizure-modulating brain targets in order to achieve a gradual, more or less continuous drug release directly into the target region of the epileptic network [43,44]. Although the capacity of such carriers is limited, controlled-release polymers have reproducible release kinetics capable of releasing drugs over a period of days to years. To reduce tissue damage, drug-eluting wafers can also be surgically positioned on the brain surface (e.g., subdural placement) so that delivery occurs via transmeningeal diffusion to a neocortical seizure-initiating region of the brain. Drugs encapsulated within these polymers are protected from clearance and degradation and are released by a combination of diffusion and polymer degradation, which can be partly controlled by modifying the composition of the polymer [49] or by use of conductive polymers that theoretically could be combined with seizure detection systems [53].

However, three main disadvantages are associated with the use of polymer-based implants to suppress epileptic seizures. First, the drug diffuses passively through the brain parenchyma, resulting in uneven drug distribution within the target area and a limited extent of delivery [13]. Drug penetration distance within the brain parenchyma by passive diffusion depends on the compound used but typically is limited to only a few mm [49,54]. While such a diffusion range may be too small in humans, it is sufficient or even too large in animal models (see chapter 6.3). Nevertheless, the first proof-of-principle of a focal drug delivery approach in man utilized the subdural application of gel foam soaked with the local anesthetic lidocaine adjacent to epileptogenic zones and revealed decreased spike counts in three patients with sysDRE [55]. Second, a sufficiently long lifespan of the implant to ensure long-term seizure control cannot be reached with polymer-based systems. Third, continuous intracranial drug delivery can be associated with the development of pharmacological tolerance [56,57], favoring techniques that allow discontinuous drug release. For the treatment of brain tumors, polymer-based implants are in further development to allow discontinuous drug release. For example, microchips containing individual drug-containing reservoirs that may allow pulsatile drug release by modifying the composition of the reservoir-covering membranes are under investigation [49].

Another strategy for continuous intracranial drug delivery is the use of external or (subcutaneously) implantable drug pumps connected to a (stereotaxically implanted) catheter/cannula system located in the ventricular system or specific targets within the brain parenchyma. Osmotic pumps connected to a catheter system also have the disadvantage of releasing the drug by passive diffusion [58,59,60,61]. Active pressure microinfusion pumps are advantageous in this respect. A recent clinical study successfully utilized a subcutaneously implanted microinfusion pump connected to a catheter system for long-term infusion of the broad-spectrum ASD valproic acid (valproate) into the ventricle of patients with sysDRE [62]. From the ventricles, the drug is distributed to the epileptic network within the brain parenchyma also via passive diffusion. Within the extracellular environment of the brain parenchyma, a drug can move either through passive diffusion along concentration gradients or by bulk flow along pressure gradients (see below). Drug distribution via diffusion within the brain parenchyma largely depends on several factors, including size, molecular weight, and polarity of the drug. For this reason, pressure techniques are highly attractive when targeting this CNS compartment. In contrast to passive diffusion obtained by implantable polymers and osmotic pumps or by icv delivery, convection-enhanced delivery (CED) of drugs directly into the brain parenchyma uses a pressure gradient produced at the tip of an infusion catheter to push the drug into the extracellular space by bulk flow [51].

The intention of CED is to distribute the drug at higher concentrations, more evenly, and over a larger range than when administered by diffusion alone [13,49,50,51]. The distribution range achievable by CED is determined by several variables, including infusion rate, tissue characteristics, cannula size, the volume of infusion, as well as by balance of bulk flow, diffusion, and clearance. Because CED relies on bulk flow (combined with diffusion) of the therapeutic compound through the extracellular space, it is not only attractive for delivery of small-sized ASDs, but also for slowly diffusing substances of high molecular weight.

Risk factors or complications associated with the direct delivery of drugs into the brain must be thoroughly investigated for each infusate to be tested. While toxic effects by a mild increase in intracranial pressure during CED are thought to be minimal, neurotoxicity from direct effects of drugs on neurons and glial cells are more relevant and may exclude some drugs or dictate dose and infusion rate [50]. Furthermore, gliosis around the cannula is frequently observed in response to chronic implantation. The disadvantages of pump–catheter systems also include catheter misplacement and the risk of reflux that may occur at high infusion rates, as the infusate travels along the catheter itself instead of into the brain parenchyma [63]. A large field of research deals with the ongoing development of CED of drugs to the CNS. A challenge for using CED to treat focal epilepsies is to restrict delivery of the therapeutic agent to the intended brain area because prolonged infusions using conventional delivery catheters or cannulas can be associated with leakage and unwanted drug distribution beyond the infusion zone. This could cause a loss of efficacy or occurrence of neurological adverse effects, respectively [13,56]. In addition, the stability of the therapeutic drug for prolonged periods at body temperature should be verified [56].

Extensive preclinical and clinical experience exists with CED of drugs to treat brain tumors and neurological diseases such as Parkinson’s disease [64] and have helped to establish CED as one of the most promising strategies for the targeted treatment of CNS disorders. CED of drugs is also intensively investigated preclinically by many groups, including ours [13,56,57], and clinically [16], to treat epilepsies. In their clinical safety study, Heiss et al. [16] showed that CED of the GABA_A_ receptor agonist muscimol for 12 to 24 h into the epileptic focus of patients with DRE did not damage adjacent brain parenchyma or adversely affect seizure surgery outcome. As mentioned above, continuous intracranial drug delivery can be associated with the development of pharmacological tolerance [56,57], thus favoring techniques, which allow discontinuous drug release [65], such as programmable drug pumps allowing intermittent drug infusion.

Furthermore, the development of intracranial drug administration only when needed, i.e., timed delivery in relation to seizure activity (closed-loop system, [66]) will probably prevent the development of tolerance and additionally reduce the risk of adverse effects caused by continuous drug flow into the brain. A proof-of-concept rat study, in which an external spike/seizure detection unit was used to trigger an external drug infusion pump, was provided by Stein et al. [67]. Further developments of this approach aimed to construct devices where both seizure detection and drug-releasing units are implantable [48,68]. The subdural pharmacotherapy device (SPD), and the subdural hybrid neuroprosthesis (HNP) as its predecessor, have been investigated preclinically as implantable devices to treat intractable focal neocortical epilepsy. They were implanted above the seizure focus of the neocortex and periodically delivered the respective drug transmeningeally via the subdural/subarachnoid space [47,69]. Cerebrospinal fluid (CSF) removal of local inflammatory cells and molecules is additionally realized, which otherwise may clog the drug delivery system [47,69]. Neural activity recordings from the treated epileptogenic area provided feedback for the electrophysiological effects of drug pulses [47,48,69]. An advantage is that such a subdural device does not damage underlying brain tissue by penetrating cannulas or catheters. However, a challenge for further development of a responsive (closed-loop) SDP system is to ensure that the delivered drug diffuses into the neocortical seizure focus fast enough to abort a developing electrographic seizure before it progresses into a clinically significant ictal episode [47,69]. Salam et al. [68] presented an implantable closed-loop device for intracerebral electroencephalography (EEG) data acquisition and seizure detection with simultaneous localized ASD injection feedback and tested the device on human EEG recordings.

Although invasive techniques are associated with several risks of complications such as mechanical device failure, catheter obstruction, catheter misplacement, infection, rejection, CSF leak, and the need for refill [49,62], highly promising results emerged from studying the concept of intracranial drug delivery in epilepsies. Nevertheless, to avoid the risk of surgical complications associated with intracranial drug delivery, it is important to consider alternative, less invasive routes of ASD administration in sysDRE. For example, the further development of iv-administered targeted drug-loaded nanocarriers constructed to facilitate BBB penetration [70], as well as the systemic administration of inactive prodrugs that are activated at the site of the seizure focus [46], may be advantageous in this respect. Further strategies include the intranasal delivery of appropriate nanocarriers or drug formulations to target a seizure focus in the hippocampal/parahippocampal network. The nasal spray delivery of the benzodiazepines midazolam and diazepam is approved for the acute treatment of seizure clusters aiming to provide emergency intervention by a fast and more direct targeting of the hippocampus via the lateral olfactory tract. Intranasal drug delivery has the advantage of being a noninvasive approach. However, this on-demand drug administration requires largely undisturbed consciousness in patients. We do not further consider these approaches in our review because they do not belong to the intracranial drug delivery approaches, which are in development for long-term seizure suppression in sysDRE.

## 3. Animal Model Aspects

In most preclinical studies on epilepsies, different types of rodent models are used. A general overview of seizure and epilepsy models is given, for example, by [71,72,73]. Apart from less frequently used genetic models for specific types of epilepsy, models of induced seizures or epilepsy are mainly used in epilepsy research, including the development of new treatment strategies. Whereas in acute models, typically used as fast screening models, seizures are induced chemically or electrically in healthy, neurologically unremarkable rodents, chronic seizure or epilepsy models are more laborious but are characterized by permanently enhanced seizure susceptibility and other enduring brain alterations that partly resemble human pathologies.

Briefly, acute seizures can be induced electrically, for example, in the maximal electroshock seizure (MES) or the 6 Hz 44 mA test, or chemically, for example, by the administration of the GABA_A_ receptor antagonists pentylenetetrazole (PTZ, metrazole). PTZ can be injected subcutaneously (sc), intraperitoneally (ip), or intravenously (iv). Further chemoconvulsants used are, for example, the GABA_A_ receptor antagonists bicuculline (ip, iv, or focally) and the volatile flurothyl. Chronic seizures or epilepsies are also induced by electrical or chemical means. Chronic seizure models of TLE can be induced, for example, by electrical kindling (e.g., via implanted electrode) of limbic structures such as the amygdala or hippocampus [74]. Chronic epilepsy models of TLE result, for example, from induction of a prolonged status epilepticus (SE) induced by the glutamate agonist kainic acid, the muscarinic agonist pilocarpine, or by sustained electrical stimulation of limbic structures, which lead, after a latency period, to the expression of spontaneous seizures [75].

Numerous different seizure/epilepsy models have been used during the past decades to investigate intracranial drug delivery approaches. However, a careful choice of the animal model may accelerate the further development of this treatment strategy for sysDRE.

First, concerning acute and chronic seizure models, those which allow reliable and repeated seizure threshold determination are advantageous because they permit easy quantification of seizure susceptibility over time in individual animals, i.e., before, during, and after (long-term) drug delivery (each animal serves as its own control). For example, the determination of electrographic or motor seizure thresholds in kindled rats as a chronic seizure model of TLE [74,76,77] as well as the determination of myoclonic and tonic seizure thresholds in the timed intravenous PTZ seizure threshold (ivPTZ-ST) test as an acute model [73,78,79,80] make use of this advantage. A further advantage of seizure threshold models is that the detection of anticonvulsant, as well as proconvulsant effects, is possible. In contrast, fixed-dose administrations of convulsants have the disadvantage that possible antiseizure effects may be overseen in individual rats, in which a fixed-dose could be overwhelming with regard to individual seizure susceptibility.

Second, models of DRE such as TLE models or models of refractory neocortical epilepsies may better predict the seizure-controlling outcome of intracranial drug delivery approaches in humans, although this is not yet known. The kindling model of TLE is well-known to be highly predictive of at least systemic drug efficacy against focal limbic seizures in humans [71]. Especially focal, but also the focal to bilateral tonic-clonic seizures in kindled rats are considered difficult to suppress, so that amygdala-kindling was the first proposed animal model of drug-resistant focal epilepsy [81]. The tetanus toxin rat model was suggested to resemble a model of refractory neocortical epilepsy [82]. Refined models of refractory TLE include models with seizures intrinsically resistant to some systemically applied ASDs (e.g., acute 6 Hz 44 mA psychomotor seizures and lamotrigine-resistant kindled rats), models where the resistance develops over time (e.g., PTZ seizure test in epileptic rats), and models based on the selection of systemic ASD nonresponders (e.g., phenytoin-resistant amygdala-kindled rats or phenobarbital-resistant post-SE model of TLE) (overview by [72]). Selection of responders and nonresponders mirrors the clinical situation that epilepsy patients exhibit a heterogeneous ASD responsiveness. However, efficacy studies of intracranial pharmacotherapy so far did not preselect systemic drug responders and nonresponders, as this is a highly time-consuming procedure. Needs to be elaborated on, intracerebral drug delivery also yields responders and nonresponders as was shown by chronic intrasubthalamic microinfusion of the ASD vigabatrin [56] and the GABA_A_ receptor agonist muscimol [57] by using the ivPTZ-ST fast screening model. It is unknown, if there is an overlap between systemic drug responsiveness and intracerebral drug responsiveness in individual animals.

## 4. Targeting the Seizure Focus

Directly targeting the seizure focus or areas close to the seizure focus with intracranial drug delivery is an obvious approach. Accordingly, numerous studies have addressed this strategy, aiming to re-establish a balance between excitation and inhibition within the abnormally excitable seizure focus and associated epileptic network. The seizure focus can be targeted directly by intraparenchymal drug delivery, indirectly by transmeningeal diffusion of an appropriate compound administered to the epidural or subdural (subarachnoid) space, or indirectly by drug delivery into the ventricular system (Figure 2).

### 4.1. Intracerebroventricular (Icv) Drug Delivery to Modulate Seizure Focus

Injections or infusions of drugs into the CSF via icv or intrathecal delivery in order to bypass the BBB is clinically established and has been used for several decades to treat different CNS disorders such as brain tumors, infectious meningitis, and intractable pain [83]. The icv route has also gained increasing interest for the treatment of sysDRE. The idea is to obtain high intracerebral anticonvulsant drug concentrations while reaching low plasma levels, thereby reducing peripheral toxicity.

Advantages of drug infusions into the ventricular system include the relatively easy access and the lack of need to clearly localize the epileptic focus [18]. However, rapid CSF turnover and the fact that CSF bulk flow is directional with a variable velocity can affect drug distribution [49,50]. Furthermore, the CSF is separated from the brain parenchyma by the ependymal layer and the glia limitans. The drug must cross the CSF-brain barrier, which has a much smaller surface area than brain capillaries and may restrict diffusion [49]. Subsequently, the drug is further distributed within the brain parenchyma by passive diffusion. Clearance from the CSF or via brain capillaries as well as drug metabolism and uptake by neurons and/or glia are further factors affecting drug distribution into seizure-modulating networks. Additionally, drugs showing a fast clearance and/or low diffusion range show a more uneven distribution in the brain [18]. The degree to which the drug crosses into the peripheral circulation may affect the risk of peripheral toxicity, while diffusion into brain areas not involved in seizure modulation may cause neurological adverse effects [18]. Thus, icv administration is not as targeted as direct delivery of compounds into specific brain regions.

#### 4.1.1. Acute Icv Drug Delivery in Animal Models of Seizures/Epilepsies

Despite the disadvantages mentioned above, animal studies utilizing acute icv injection of different types of compounds, including ASDs in different seizure and epilepsy models, suggested that the delivery of drugs into the CSF may be an alternative route in the treatment of sysDRE. In these acute icv injection studies, a wide range of drugs with GABAergic and non-GABAergic mechanisms of action have been shown to have antiseizure potential in different preclinical models, e.g., GABA uptake inhibitors in the acute scPTZ seizure test in mice [84]. Mixed anticonvulsant and proconvulsant actions were observed with icv injection of GABA uptake inhibitors and with muscimol in ivPTZ-induced and isonicotinic acid hydrazide-induced seizures in rats and in the ivPTZ-ST test in rats [85]. The ASDs phenobarbital, phenytoin, midazolam, and valproate were anticonvulsant in the MES test in rats [86], and the ASDs phenobarbital, carbamazepine, and phenytoin in the amygdala-kindling model in rats [87]. The ASD levetiracetam, but not phenytoin, suppressed 6 Hz 49 mA-induced seizures in mice [88]. Midazolam and allopregnanolone were effective against picrotoxin-induced seizures in mice [89], and histamine H3 antagonists against amygdala-kindled and MES in rats [90]. The anticonvulsant potential was also shown by icv injection of valproate, and orexin receptor antagonists on PTZ-induced kindled seizures in rats [91,92], the gap junction blockers quinine and carbenoxolone on penicillin-induced epileptiform activity [93,94], and ant venoms on seizures induced by icv injection of bicuculline [95].

However, for long-term symptomatic seizure suppression, chronic infusion of appropriate substances into the ventricle system is inevitable. Furthermore, depending on physicochemical properties of the drug, such as liposolubility and ionization at local pH, not every drug penetrates deeply into the brain parenchyma after acute icv injection. This is the reason why high doses are often necessary to reach antiseizure efficacy with bolus icv injections. Thus, an acute bolus may be more toxic and less effective than small icv doses delivered repeatedly or continuously [96]. The occurrence of neurological adverse effects after bolus icv injection, therefore, does not necessarily mean that a certain drug is unsuitable for chronic icv infusion. Nevertheless, a thorough investigation of (neurological) adverse effects and of risks for pathological impacts induced by chronic icv drug infusions is required for each drug of interest.

#### 4.1.2. Chronic Icv Drug Delivery in Animal Models of Seizures/Epilepsies

In a comprehensive study, acute ip, acute icv, and chronic icv (7 days via osmotic pump) administration of valproate was investigated using the amygdala-kindling rat model of TLE to directly compare efficacy, toxicity, and drug levels between the three different administration routes [58]. At doses suppressing focal and generalized kindled seizures, acute ip and acute icv injections were shown to be accompanied by remarkable ataxia and sedation in addition to piloerection and wet-dog shake behavior after acute ip injection and pronounced contralateral hemiparesis or hemiplegia after acute icv injection. In contrast, chronic icv infusion controlled focal and generalized seizures without producing remarkable adverse effects, except for some mild ataxia, the most effective dose being described as 0.8 mg/h (i.e., 19.2 mg/d) [58]. Mean brain concentration after chronic icv injection was 123.2 µg/g, while plasma or hepatic drug concentrations were much lower [58], thus avoiding potential systemic adverse effects. Indeed, plasma levels were considerably low after chronic icv infusion (23 µg/mL) in the study by Serralta et al. [58] compared to 640 µg/mL after chronic (repeated) ip administration of valproate at 200 mg/kg 3 times daily for 6 weeks [97], the latter of which induced similar anticonvulsant effects, but more adverse effects. Brain concentrations of valproate were 144 to 187 µg/g in the study by Löscher et al. [97].

A pathological and behavioral study in pigs (ventricular ependyma comparable between humans and pigs) indicated behavioral adverse effects at doses higher than three mg/day of the ASD phenytoin and higher than 1.5 mg/day of valproate chronically infused icv [98]. However, they did not reveal damage to the brain in response to several weeks of icv infusion of the two ASDs [98], suggesting that icv infusion of those drugs could be well tolerated by humans. Noteworthy, a study by Walrave et al. [88] indicated proconvulsant actions of a surgically implanted icv cannula targeting the left ventricle in mice, which was suggested to be caused by potential inflammatory processes induced by cannula implantation, emphasizing the need for thorough risk assessments of intracranial drug delivery techniques.

Oommen et al. [99] infused the ASD gabapentin for five days icv by means of osmotic pumps and observed anticonvulsant effects in the acute flurothyl seizure model. There were no measurable serum levels of gabapentin, and experiments with methylene blue showed dye in periventricular white matter and in the cortex [99]. Chronic icv drug delivery was also investigated by implanting an adenosine-releasing polymer unilaterally into the lateral ventricle [100]. Cardiovascular adverse effects limit the systemic administration of the nucleoside adenosine. The release time for icv delivery was 17 days. The endogenous inhibitory neuromodulator adenosine exerted transient (one week) anticonvulsant activity in hippocampus-kindled rats by lowering seizure severity and reducing seizure duration. One important question is if the brain structures relevant for modulation of specific seizure or epilepsy types are accessible for a certain drug administered icv. For example, it was suggested that some systemically active anticonvulsants act to suppress seizures induced by scPTZ at a site not readily affected by icv injection [84]. Nevertheless, the preclinical studies showed that acute and chronic icv infusions of ASDs and other compounds could control seizures, but neurological adverse effects are often not improved compared with systemic delivery. However, systemic toxicity is likely to be reduced due to low plasmatic and hepatic drug levels after icv infusion.

#### 4.1.3. Icv Drug Delivery in Humans with Drug-Resistant Epilepsy (DRE)

A first-in-man clinical study recently investigated chronic (several months), continuous unilateral icv infusion of valproate in patients suffering from drug-resistant focal seizures with or without secondary generalization [62]. Valproate is generally considered well-tolerated, and hepatotoxicity is rare, but its teratogenicity limits its chronic use in pregnant women, and other systemic adverse effects are frequently described. The study focused on dose-finding, pharmacokinetics, and safety of this approach [62]. Five TLE patients in which systemic valproate was ineffective were included in this study and were implanted sc with a pump connected to a catheter system. The findings indicate anticonvulsant effectiveness and improved quality of life despite some adverse effects such as nausea and appetite loss. There was no evidence of local periventricular toxicity. High CSF levels were achieved with corresponding low serum levels [62]. In a next step, a clinical phase 2 study is currently recruiting patients with medically refractory focal seizures with temporal lobe onset with or without secondary generalization to assess the safety and anticonvulsant efficacy of icv delivery of a reformulation of valproate [101]. The drug is again delivered icv via an implantable drug pump system. The study is conducted as a double-blind, randomized, placebo-controlled trial.

In summary, icv delivery is a promising approach to achieve anticonvulsant effects while reducing systemic toxicity but does not necessarily improve neurological toxicity due to unwanted distribution of the drug into areas not involved in the epileptic network in addition to the wanted diffusion to epileptogenic areas.

### 4.2. Transmeningeal Drug Delivery to Modulate Seizure Focus

Apart from icv delivery of drugs, compounds with antiseizure activity can be applied to the brain surface via implantation of drug-releasing catheter systems or cups/carriers into the epidural or subdural (subarachnoid) space overlying a neocortical seizure focus. The concept of drug delivery through spinal meninges is clinically established for the administration of local anesthetics and analgesics, for example, during obstetric pain relief or lower extremity surgery. Concerning intracranial drug delivery, the transmeningeal approach has the advantage of being less invasive than direct intraparenchymal or icv drug delivery, but it requires seizure-suppressing molecules that can penetrate the meninges and diffuse into the underlying neocortex in sufficient amounts. Epileptic foci located deeper in the brain parenchyma are not sufficiently reached by this approach [102]. Therefore, this approach is mainly applicable for intractable focal neocortical epilepsies. Ludvig et al. [103] showed that a short-term 1 h epidural exposure of 1 mM muscimol is able to diffuse transmeningeally to the neocortical tissue in a spatially controlled manner in rats, thereby supporting the rationale of using transmeningeal drug delivery for the treatment of intractable focal neocortical epilepsy. Nevertheless, the risk of altering neocortically processed sensory, motor, and cognitive functions by long-term transmeningeal drug delivery must be thoroughly assessed for each compound of interest.

#### 4.2.1. Acute Transmeningeal Drug Delivery in Animal Models of Seizures/Epilepsies

In a series of early proof-of-principle studies, acute epidural or subdural administrations of different compounds were shown to suppress chemically induced neocortical epileptiform activity in a concentration-dependent manner. Muscimol applied topically to the cortex in rats suppressed neocortical epileptiform activity induced by penicillin, bicuculline, and picrotoxin, albeit not strychnine [104]. Acute epidural bolus injections of the ASD diazepam reduced acute epidural bicuculline-induced neocortical seizure spiking in rats [105]. The group of Ludvig showed that epidural pentobarbital [106] and muscimol [107,108] delivery was able to prevent neocortical seizures locally induced by epidural administration of acetylcholine in rats. The drugs were applied for several minutes by an epidural cup implanted over the neocortex. However, GABA was not able to prevent the acetylcholine-induced neocortical seizures but acutely terminated them once they have been induced [109]. This may be due to reduced responsiveness of GABA_A_ receptors upon continuous exposure to GABA, thereby not only inducing tolerance but also increasing the risk of GABA withdrawal seizures, similar to what is described for intraparenchymal GABA delivery [110,111]. In a comparative study with epidural cup delivery of drugs at a fixed concentration of 1 mM aiming to prevent disturbance of physiological osmolarity of CSF and brain extracellular fluid, it was shown that only muscimol, but not lidocaine, midazolam, pentobarbital, and GABA, exerted anticonvulsant effects in this rat model [108]. Furthermore, local physiological multineuronal activity may be undisturbed at least by short-term transmeningeal muscimol treatment [112].

#### 4.2.2. Chronic Transmeningeal Drug Delivery in Animal Models of Seizures/Epilepsies

Again, chronic continuous or discontinuous drug delivery is necessary for long-term seizure suppression. In a chronic transmeningeal drug delivery study, Tang et al. [65] showed that discontinuous (50 µL once per day for four consecutive days in each week) epidural muscimol (1 mM) delivery over three weeks maintained antiseizure efficacy without inducing tolerance in rats, which we showed to otherwise occur after about two weeks of continuous intraparenchymal muscimol delivery [57]. In a series of further experiments, Ludvig and colleagues refined their above-described experiments of transmeningeal drug delivery by developing the SPD (see chapter 2). This implantable drug delivery system involves a radio-frequency-controlled dual minipump. It allows chronic (periodic) drug delivery and CSF removal via subdural/subarachnoid strips equipped with fluid-exchange ports and simultaneous recording of drug effects on neural activity from the treated seizure focus [47,48,69]. Using this device, Ludvig et al. [113,114] performed safety studies of chronic (several months) discontinuous subdural/subarachnoid muscimol (1 mM) delivery in nonhuman primates and showed that the device was well-tolerated for up to 11 months without indication of altered motor performance and spatial memory, and without changing body weight. There were no detectable levels of muscimol in the blood and cisternal CSF, but protein levels in the cortical-site CSF were significantly higher than normal [113]. Seizure-preventing effects were not investigated in this study, but an earlier investigation showed that muscimol delivered acutely into the subarachnoid space exerts a focal seizure–preventing effect in the nonhuman primate neocortex [107].

Drug-loaded polymers also have been investigated using the transmeningeal route. Halliday et al. [102] subdurally implanted biodegradable polymer sheets loaded with the ASD levetiracetam above the motor and somatosensory cortices of chronic epileptic rats with hippocampal tetanus toxin-induced seizure focus. Seizure duration was shortened, while seizure frequency was not significantly reduced by this approach, indicating that levetiracetam may not have reached the hippocampal seizure focus in sufficient amounts 4 to 5 mm away from the polymer implant site [102].

#### 4.2.3. Transmeningeal Drug Delivery in Humans with DRE

Because epidurally or subdurally located drug-releasing polymer systems are highly limited in drug load capacity, a frequent refill or exchange would be necessary for long-term transmeningeal drug delivery. The success of transmeningeal pharmacotherapy in epilepsies thus argues for drug delivery on demand in closed-loop systems and/or the use of subcutaneously implanted microinfusion pumps with larger drug reservoirs connected to appropriate catheter systems. Nevertheless, in a first-in-man study on intracranial drug delivery in epilepsies, a lidocaine-soaked gel foam was placed for several minutes onto the pia mater above the seizure focus of three patients prior to tissue resection, and within minutes a clear reduction in interictal EEG spike activity was detected [55]. Because of considerable experience with lidocaine in spinal-epidural anesthesia, this compound was used to explore the viability of transmeningeal drug delivery in this first proof-of-principle clinical trial on intracranial drug delivery approach in epilepsies.

### 4.3. Intraparenchymal Drug Delivery to Modulate Seizure Focus

The most direct and obvious approach to deliver a drug to its target in the brain is to administer it directly into the parenchyma. Compensating local deficits in inhibition or reducing hyperexcitation can be considered as a restorative network modulation. Mainly the epileptogenic zone of the brain will be modulated, thereby minimizing neurological toxicity in addition to the lowered risk of systemic adverse effects. Depending on the used technique, the compound diffuses passively (acute bolus injection, osmotic pumps, and implantable sustained drug-releasing polymers) or convection-enhanced (microinfusion pumps connected to catheter system) through the brain interstitium. In general, preclinical studies indicate that intrafocal drug delivery approaches are well tolerated, although behavioral toxicity was not investigated in many of the studies (see below). The occurrence of slight adverse effects may be related to the used drug and can be partially explained by drug diffusion into neighboring brain areas. Likewise, observed anticonvulsant effects are probably not always induced solely by modulation of the seizure focus, but also by infiltration of adjacent seizure-modulating areas.

#### 4.3.1. Acute Intraparenchymal Drug Delivery in Animal Models of Seizures/Epilepsies

Kelso et al. [115] directly compared the efficacy and tolerability of systemic versus intrafocal ASD delivery in a model of refractory cortical epilepsy in rats. Acute microinjection of phenytoin, but not tiagabine, into the cortical focus of the tetanus toxin model of cortical epilepsy reduced behavioral and EEG seizures without obvious behavioral adverse effects, while high sedative doses of systemic phenytoin did not affect seizures [115]. This study is the first proof-of-principle that focal drug delivery can be more effective than systemic drug administration in a model of pharmacoresistant epilepsy. However, another study showed that focally evoked pilocarpine-induced seizures were completely prevented by systemic vigabatrin premedication, but the rats were only partly protected by acute intrahippocampal delivery of the drug [116]. Successful intrafocal drug delivery is likely dependent on the choice of drug, focus localization, drug distribution range, and animal model.

Indeed, there is a considerable amount of preclinical studies providing evidence for anticonvulsant activity by direct delivery of many different drugs with a variety of mechanisms of action into or close to the seizure focus [18]. Many of the studies also helped to better understand seizure generation and propagation mechanisms. Acute intrafocal (intrahippocampal) diazepam reduced ictal spiking in the focal cobalt/pilocarpine and in the focal bicuculline seizure model, without measurable systemic levels of diazepam in most animals [105,117]. In an earlier study, Piredda and Gale [118] showed that acute microinjection of muscimol into the anterior piriform cortex region (area tempestas) prevented seizures induced by local injection of bicuculline, kainic acid, glutamate, and carbachol, whereas local administration of the muscarinic antagonist atropine only prevented seizures induced by carbachol.

Acute microinjection of lidocaine into a bicuculline-induced seizure focus in the piriform cortex suppressed electrographic and behavioral seizures in rats [119]. The intrafocal injection of other non-GABAergic compounds including taurine [120], a group II metabotropic glutamate receptor agonist [121], excitatory amino acid receptor antagonists [122,123], dopamine D2 receptor agonists [124], adenosine analogs [125,126,127], and the calcium channel blocker nimodipine [128] has been effective in seizure/epilepsy models. Acute microinjection of gap junction blockers into the cortical epilepsy focus of the tetanus toxin model [129] or into the amygdala in amygdala-kindled rats [130] exerted anticonvulsant effects. Botulinum neurotoxin E microinjected into the hippocampus inhibited glutamate release, reduced focal and generalized kainate-induced seizures and prevented neuronal loss and long-term cognitive deficits associated with kainic acid seizures [131]. Acute intrahippocampal injections of oxytocin and diazepam [132], the peptide hormone ghrelin [133], and orexin receptor antagonists [134] induced anticonvulsant effects in the acute single-dose PTZ seizure model.

In addition, acute bolus delivery of arsono analogs of GABA and aspartate, but not the arsono analog of glutamate, into the amygdala exerted anticonvulsant effects by elevating afterdischarge thresholds in amygdala-kindled rats [135,136]. The metabotropic glutamate receptor agonist L-2-amino-4 phosphonobutyrate (L-AP4) raised the generalized seizure threshold in amygdala-kindled rats after focal administration, probably due to inhibition of presynaptic glutamate release [137]. The group also showed that 2-chloroadenosine, a nonmetabolizable adenosine A1 receptor agonist, is able to raise kindled seizure thresholds in rats after intra-amygdalar microinjection [138]. Anticonvulsant effects on amygdala-kindled seizures were shown by acute intrahippocampal lidocaine delivery [139] and by acute delivery of the N-methyl-D-aspartate (NMDA) receptor antagonist 2-amino-5 phosphonovalerate (APV) into the perirhinal cortex [123], i.e., epileptic network areas closely connected to the seizure generation zone (Figure 1) and involved in seizure propagation without directly targeting the seizure focus. Likewise, acute microinjection of GABA into the amygdala attenuated the expression of secondarily generalized seizures induced by the kindling of the insular or entorhinal cortex [140].

Systemic pilocarpine-induced seizures were prevented by microinjection of the NMDA receptor antagonists 2-amino-7-phosphonoheptanoate (APH) into the piriform cortex [141]. Local increase of GABA by acute delivery of the irreversible GABA degradation inhibitor vigabatrin into the piriform cortex was anticonvulsant against systemic bicuculline-induced seizures but ineffective against seizures induced by maximal electroshock [142]. Furthermore, vigabatrin microinjected into distinct subregions of the piriform cortex raised seizure thresholds in fully amygdala-kindled rats but also induced circling behavior and/or mild ataxia, a change of motor behavior or stereotypies in half of the rats [143,144]. In addition, part of the rats showed initial proconvulsant effects, which has also been observed after systemic vigabatrin injection [145]. Nevertheless, the studies emphasize the critical role of (specific subregions of) the piriform cortex in the generation, amplification, and propagation of forebrain (limbic type) seizures [146]. Indeed, amygdala-kindling in rats was shown to induce persistent changes in firing rate and glutamate sensitivity [147], as well as a decreased number of GABAergic neurons in the ipsilateral central piriform cortex [148]. Likewise, in human patients, increased cerebral blood flow and reduced GABA_A_ receptor binding in response to frequent seizures have been found near the frontal piriform cortex ipsilateral to the presumed cortical focus, irrespective of where interictal or ictal activity occurs in the cortex [149]. This area of the human primary olfactory cortex, therefore, has been suggested to be an attractive target for epilepsy therapy, including neurosurgery, electrical stimulation, and targeted drug delivery [149].

#### 4.3.2. Chronic Intraparenchymal Drug Delivery in Animal Models of Seizures/Epilepsies

Again, acute intracerebral drug applications have a limited effect duration. To prolong the effect of a drug on the seizure focus, delivery from implantable osmotic pumps, polymer carriers, and chronic CED has been investigated.

Kohane et al. [150] directly compared acute intrahippocampal injection of a free muscimol solution with intrahippocampal placement of muscimol-loaded lipid–protein–sugar microparticles into the hippocampus before induction of acute intrahippocampal pilocarpine-seizures. Both approaches mitigated seizure onset, but the results strongly indicated that encapsulation enhanced the protective antiseizure effect of muscimol. Furthermore, pilocarpine-induced histopathological changes were slightly less pronounced with encapsulated compared to free muscimol [150]. Muscimol, which in the brain is degraded much more slowly than GABA, was administered 80 min before the end of the pilocarpine infusion. The authors suggested that free muscimol may have largely diffused away from the site of injection during that interval, whereas the encapsulated form may have maintained an effective concentration for a longer time [150].

While GABA itself is not appropriate for systemic administration because of its low ability to pass the BBB, this property is of interest for intracerebral infusion. Continuous (7 days) intracortical [110] and intra-amygdalar [59] GABA infusion via osmotic minipumps connected to implanted cannulas induced anticonvulsant effects in amygdala-kindled rats. However, this strategy may be associated with the risk of GABA withdrawal seizures upon stopping infusion [110]. Kokaia et al. [151] showed that noradrenaline released from polymer matrices implanted into the hippocampus failed to retard the development of hippocampal kindling in noradrenaline-depleted rats, while GABA-loaded polymer matrices placed directly above the substantia nigra pars reticulata (SNr) prevented generalized seizures in amygdala-kindled rats ([151]; refer to chapter 5.1.3).

Polymeric microspheres loaded with phenytoin and injected into the hippocampus caused anticonvulsant effects in the rat tetanus toxin model of epilepsy [152]. Likewise, the delivery of phenytoin from controlled-release macroscopic polymers implanted in the cortical seizure focus reduced seizures in a rat model of cobalt-induced epilepsy [153]. Implantation of a titania bioceramics reservoir containing valproate into the amygdala of chemically kindled rats suppressed seizures [154]. Neocortical valproate-containing polymer implants significantly enhanced survival in rats with tetanus toxin/cobalt neocortical epilepsy focus [155] and reduced epileptiform potentials [156].

(Neuro) peptides only show limited capacity to pass physiological barriers after oral or iv injection. The neuropeptide thyrotropin-releasing hormone (TRH), known to exert anticonvulsant effects, showed prolonged anticonvulsant activity in response to sustained release from polymeric microdisks implanted into the amygdala in the rat amygdala-kindling model [157]. The nucleoside adenosine exerted anticonvulsant effects for 10 days during kindling progression and in fully kindled rats in response to release from silk polymers implanted into the infrahippocampal cleft in the rat hippocampal kindling model [158,159]. Continuous infusion of adenosine over two weeks via osmotic minipumps connected to cannulas implanted into the hippocampus of kainate-induced epileptic rats reduced spontaneous seizure frequency without inducing adverse effects [60].

Continuous CED of high-molecular-weight ω-conotoxins, acting as irreversible presynaptic N-type calcium channel antagonists, into the amygdala of rats via an external pump over 20 min induced long-lasting (about 1 week) anticonvulsant effects on amygdala-kindled electrographic seizures and thresholds, whereas intra-amygdalar carbamazepine induced only short-term anticonvulsant effects [160]. Noteworthy, after intra-amygdalar delivery of ω-conotoxins, a tremor was observed in some rats only in response to high doses, whereas icv injections of these compounds caused locomotor arrest and whole-body tremors [160]. Comparably, 20 min intraamygdalar delivery of botulinum toxins A and B caused long-term anticonvulsant effects for up to 50 days in the same rat model without inducing obvious adverse effects [161]. However, botulinum toxin B caused a delayed proconvulsant action as well as spontaneous and handling-evoked behavioral seizures when infused into the hippocampus in the ivPTZ-ST rat model [162]. This may be due to the ability of botulinum toxin B to modulate both excitatory and inhibitory terminals and, in addition, may be related to the difficulty to therapeutically modulate the highly complex hippocampal network and its changes associated with epilepsies [29,31]. Targeting less complex structures may be advantageous in this respect (see chapter 5.1).

Stein et al. [67] were the first who preclinically investigated a closed-loop drug delivery system in a seizure model. Using an external programmable infusion pump, diazepam was directly injected into the bicuculline-induced seizure focus of rats after computerized detection of seizures. Anticonvulsant effects were observed by this approach [67], which minimized unnecessary drug infusion. Mangubat et al. [163] combined direct, responsive therapeutic neurostimulation of afferent hippocampal white matter pathways and on-demand intrahippocampal CED of the ASD carisbamate in a bihemispheric self-sustained focal-onset epilepsy model in rats. This double closed-loop approach significantly decreased electrographic seizure frequency. Noteworthy, intracerebral pulsatile carisbamate delivery showed stronger anticonvulsant effects than closed-loop direct stimulation therapy alone [163].

Heiss and colleagues published a series of studies in which they investigated CED of muscimol into the brain parenchyma of nonhuman primates [164,165,166], which then resulted in a first clinical trial [16]. The local distribution, toxicity, and safety of regional selective neuronal suppression by prolonged (several days) unilateral hippocampal CED of muscimol using an integrated catheter electrode and an external pump revealed that, depending on drug concentration, neurological function was normal or expressed as reversible apathy and somnolence in rhesus monkeys [166]. The gray matter of the hippocampus, amygdala, and cortex of the ipsilateral temporal lobe were the main distribution sites compared with adjacent white matter tracts. The group then determined if CED of muscimol could be monitored by real-time magnetic resonance imaging (MRI) [165]. Tritiated muscimol and the surrogate MRI tracer Gd-diethylenetriamine pentaacetic acid (DTPA) were co-infused over about 8 to 40 min into the striata of nonhuman primates. Postmortem quantitative autoradiography and histological analysis confirmed the feasibility of image-guided CED. The approach offers the possibility to control drug distribution range and to correlate distribution with clinical effects [165].

#### 4.3.3. Intraparenchymal Drug Delivery in Humans with DRE

Based on the above described preclinical feasibility experiments in nonhuman primates [164,165,166], Heiss et al. [16] recently examined the safety and effectiveness of CED of muscimol for 12 to 24 h into the brain via a depth electrode-catheter assembly to control seizures in three patients with intractable epilepsy prior to focus resection. Only one patient (cortical seizure focus) experienced reduced seizure frequency during muscimol infusion compared to vehicle infusion, while two patients with hippocampal seizure focus did not benefit from this approach. However, tracking the muscimol infusate was not successful, so that adaptions of infusion rate and duration were unfeasible. No infusion-related brain injuries were noted [16]. The study emphasizes the importance of additional preclinical studies to further develop the intrafocal drug delivery approach for epilepsies.

## 5. Targeting Remote Brain Structures of the Epileptic Network

As mentioned in the introduction, resection of the epileptogenic zone as a surgical epilepsy treatment may be effective for selected patients with sysDRE. Achieving seizure freedom by focus resection is more likely in patients with electro-clinically concordant structural lesions in the temporal lobe, while the expected outcome is less successful for patients with extra-temporal lobe foci, focal-to-bilateral tonic-clonic seizures, inconspicuous imaging findings, and occurrence of psychiatric co-morbidities [11]. However, for many patients with sysDRE, focus resection is not an option at all due to a poorly localized or undefinable seizure focus, the occurrence of a mirror focus in the contralateral hemisphere, the existence of multiple seizure foci, unacceptable surgical risks, or expected unwanted postoperative adverse effects. Apart from directly targeting the seizure focus and thereby being confronted with limitations similar to those associated with focus resection, another highly promising strategy is to deliver appropriate drugs into brain regions crucially involved in seizure propagation and remote modulation of seizure initiation (Figure 1 and Figure 2). A number of experimental studies involving animal models of seizures and epilepsy as well as clinical studies in humans have identified different specific subcortical anatomical sites critically involved in the pathogenesis, propagation, and control of seizure activity [12,32,36,37,38,39,41,167,168,169]. Indeed, focal seizures emanating from limbic areas do not propagate randomly through the brain but via anatomically well-defined pathways, including basal ganglia and thalamic regions (Figure 1). These brain networks remote to limbic and neocortical structures, therefore, play an important role in the control of different types of epilepsies/seizures and offer important targets for intraparenchymal drug delivery. Indeed, there is increasing support for the concept that therapeutic efficacy can be achieved through neuromodulation of seizure networks, rather than simple disruption of seizure generation [10]. Especially the basal ganglia have been suggested to be part of the epileptic network and to offer highly attractive targets for therapeutic intracranial drug delivery approaches.

### 5.1. Basal Ganglia

#### 5.1.1. Basal Ganglia Anatomical and Pathophysiological Background

As illustrated in Figure 1, the basal ganglia are a group of highly interconnected subcortical nuclei, which closely interact with the cerebral cortex and thalamus, but also with limbic circuitry including hippocampus and amygdala, and with pedunculopontine nucleus (PPN) and dorsal midbrain such as the deep/intermediate layers of the superior colliculus (SC) [170,171]. The cortico-basal ganglia–thalamo–cortical loop is topographically organized and involves parallel, segregated cortical-subcortical circuits with little convergence [170]. The basal ganglia loops are involved in motor, oculomotor, associative, and limbic functions, respectively. Despite the strong association between basal ganglia pathology and the occurrence of movement disturbances, basal ganglia are not just simply involved in online-control of movement but rather have a prominent role in higher-order behavioral and cognitive functions, including motor learning and automatic execution of movements [170]. Cortical inputs enter the basal ganglia via the dorsal striatum (caudate, putamen), the ventral striatum (nucleus accumbens, NAcc), and the STN, the latter of which being the so-called hyperdirect cortico–subthalamo–pallidal pathway (Figure 1). Processed striatal information is sent to the basal ganglia output structures internal globus pallidus (GPi) and substantia nigra pars reticulata (SNr) via two major pathways. The direct pathway is composed of monosynaptic connections between the striatum and GPi/SNr, and the indirect pathway is composed of polysynaptic connections involving connections from the external globus pallidus (GPe) to the output regions directly or indirectly via the STN (Figure 1). Most basal ganglia projection neurons utilize GABA as the main transmitter, while the STN is composed of glutamatergic neurons. Furthermore, the circuitry is modulated by dopaminergic neurons from the substantia nigra pars compacta (SNc). The striatum is a highly complex structure composed of GABAergic projection neurons and GABAergic and cholinergic interneurons. The basal ganglia output neurons of GPi and SNr exert tonic inhibitory influence onto downstream structures, including the thalamus, PPN, and the deep/intermediate layers of the SC [172,173,174]. Crosstalk between basal ganglia and limbic circuits is mediated via different structures, including cortex, NAcc, and lateral habenula [175]. In addition, anatomical connections between basal ganglia and piriform cortex have been shown [143].

Pathophysiological models of basal ganglia in epilepsies do not only include changes in firing rates and excitation/inhibition but also changes in firing patterns, including circuit-wide oscillations and burst discharges. Accordingly, in vivo, single-unit recording studies in amygdala-kindled rats modeling TLE showed seizure-outlasting plastic network changes in all basal ganglia regions investigated, including striatum [176], SNr [177,178], STN [179], and downstream PPN neurons [180]. Overall, these findings indicated that apart from long-lasting firing rate changes, an irregular neuronal discharge pattern reflects a pathological condition, while a regular pattern rather resembles a physiological or therapeutically treated condition. Furthermore, amygdala-kindled rats show reduced activity of the GABA-synthesizing enzyme glutamic acid decarboxylase (GAD), reduced nerve terminal (synaptosomal) GABA concentrations, reduced GABA receptor binding in SNr, and increased binding in the striatum [181,182]. The disturbed GABAergic activity in the basal ganglia may underlie some of the electrophysiological findings described above. While systemic pilocarpine in rats causes widespread neuronal damage in several brain regions, including SNr [183], amygdala-kindling does not induce neurodegeneration in the SNr [184], showing model-dependent neuropathological changes in the basal ganglia.

Investigations on human patients showed that the basal ganglia are hyperactive during focal to bilateral tonic-clonic seizures [185]. In a resting-state functional magnetic resonance imaging (fMRI) study with network-based analysis in 96 patients with drug-resistant TLE, He et al. [32] showed impaired inhibitory interactions within basal ganglia and between basal ganglia and thalamus and suggested that this may contribute to abnormal cortico-thalamic synchronization leading to secondary seizure generalization in TLE. Patients with TLE showed basal ganglia atrophy, which was more pronounced in patients with secondary seizure generalization [186]. Importantly, both the basal ganglia and the thalamus are involved not only during secondary seizure generalization but also during focal seizures [187]. An intracranial EEG study has reported changes in cortico-striatal synchronization level during focal seizures indicating involvement in endogenous mechanisms controlling duration and termination of abnormal oscillations [169]. Hetherington et al. [188] showed that the neuronal injury in the hippocampus of drug-resistant TLE patients was directly correlated with the neuronal damage in the putamen.

#### 5.1.2. Acute Basal Ganglia Drug Delivery in Animal Models of Seizures/Epilepsies

About 40 years ago, Gale and Iadarola [189,190] were the first who correlated an increase of GABA in the ventral midbrain with antiseizure effects. In a subsequent study, they showed that acute microinjection of muscimol and vigabatrin into the SNr suppressed tonic hindlimb extension in the MES test and blocked tonic and clonic seizures produced by fixed-dose ivPTZ and iv bicuculline in rats [191]. After this pioneering work of Karen Gale’s group on the SNr, numerous further preclinical studies performed by several different groups repeatedly confirmed and extended the finding that pharmacological inhibition of the SNr is able to suppress both behavioral and electrographic expression of many different seizures and epilepsy types. Furthermore, not only seizure propagation and generalization is prevented, but also the seizure initiation of different experimentally induced seizure types emanating from forebrain circuits is significantly impeded (reviewed by [36,39,41,167,192]. Indeed, modulation via SNr (or STN) of tonic seizures involving brainstem structures seems to be less efficient [193,194].

Most studies on intraparenchymal drug delivery targeting basal ganglia structures again were performed by acute microinjections. The SNr receives direct inhibitory (GABAergic) input from the striatum and GPe and excitatory (glutamatergic) input from the STN. Accordingly, numerous studies showed that acute bilateral microinjection of GABA-mimetic drugs such as vigabatrin and muscimol or NMDA receptor antagonists into the SNr suppresses seizures in rat models of TLE. Acute bilateral microinjection of the irreversible GABA degradation inhibitor vigabatrin [195] or the NMDA receptor antagonist APH [196] into the SNr (but not into the striatum) suppressed the occurrence of electrographic and behavioral seizures induced by ip-injected pilocarpine. Likewise, microinjection of an NMDA receptor antagonist or a partially selective antagonist at the kainate receptor into the SNr or GPi protected against electroshock and pilocarpine-induced seizures in rats [141,197]. Focally evoked pilocarpine-induced seizures were completely prevented by systemic vigabatrin premedication, but the rats were only partly protected by acute intranigral delivery of the drug [116]. This lack of robust anticonvulsant effects may be due to the fact that vigabatrin was injected only unilaterally in this study, although bilateral intranigral microinjections are necessary to prevent seizures induced by systemic pilocarpine administration [195].

Bilateral microinjection of vigabatrin into the SNr of rats suppressed amygdala-kindled seizures [198,199]. Bilateral microinjection of muscimol and vigabatrin into the SNr of rats suppressed both motor and electrographic seizures induced by kindling stimulation of different forebrain structures including stimulation of the amygdala, olfactory structures, and lateral entorhinal cortex, emphasizing the anticonvulsant properties of basal ganglia independent of the localization of the seizure focus in the forebrain [200]. Microinjections of either an NMDA receptor antagonist in the SNr or muscimol into the STN reduced amygdala-kindled motor seizures, providing evidence for the involvement of the subthalamo-nigral projection in the modulation of amygdala-kindled seizures in rats [201].

Bilateral microinjection of muscimol or the NMDA receptor antagonist 2-amino-7-phosphono-heptanoic acid (AP7) into the SNr protected against motor seizures evoked by unilateral bicuculline injections into the anterior piriform cortex [202]. Bilateral intranigral morphine did not exert anticonvulsant effects in that study, although adverse effects such as stereotyped sniffing and gnawing behavior were similar with the three investigated drugs. Bilateral, but not unilateral, microinjections of vigabatrin into the SNr dose-dependently elevated seizure thresholds induced by flurothyl [203] and PTZ [204].

Neurons in the SNr receive excitatory substance P input from the striatum. Accordingly, substance P antagonists microinjected bilaterally into the SNr also attenuated motor seizures induced by maximal electroshock or iv-injected bicuculline [205]. Further non-GABAergic substances were investigated. For example, acute bilateral intranigral opioid injections in rats drug-dependently exerted antiseizure effects in the MES test [206], in amygdala-kindled rats [207], but not in the focal bicuculline model [202]. The selective serotonin uptake inhibitor fluoxetine microinjected bilaterally into the SNr (with or without blockade of nigral GABA_A_ receptors) protected against convulsive seizures evoked by focal injection of bicuculline into the anterior piriform cortex [208].

Apart from vigabatrin, further ASDs have been tested regarding their ability to control seizures upon intranigral delivery. Midazolam protected against pilocarpine-induced secondarily generalized seizures after acute bilateral microinjection into the SNr, NAcc, and caudate-putamen [209]. Phenytoin only showed significant seizure protection after injection into caudate-putamen. In contrast, levetiracetam provided seizure protection after injection into SNr and NAcc, emphasizing the important role of appropriate drug and target choice.

Meurs et al. [210] compared systemic (ip) administration of the ASD topiramate with intrafocal (intrahippocampal) and intranigral microinjection in the focal pilocarpine model in rats. The results deserve attention because systemic as well as bilateral intranigral, but not intrahippocampal topiramate administration suppressed seizures when administered prior to intrahippocampal pilocarpine. The results suggested that the anticonvulsant site of action of topiramate is not the site of seizure induction, but one or more brain areas at a distance from this site [210]. Topiramate has multiple mechanisms of action, including increasing GABAergic inhibition, reducing glutamatergic excitation, and blocking voltage-gated sodium channels. Nigral GABA_A_ receptor blockade by picrotoxin abolished the anticonvulsant effect of topiramate in the SNr, showing that the anticonvulsant effect of topiramate was mediated via nigral GABA_A_ receptors.

Noteworthy, several ASDs administered systemically are known to reduce the firing rate of SNr neurons in naïve rats [177,211]. It was suggested that this might be an important mechanism through which some ASDs exert their anticonvulsant properties. Indeed, we could show that the anticonvulsant response to valproate in kindled rats is correlated with its effect on neuronal firing in the SNr [178]. Furthermore, providing a similar profile of action as with systemic administration, acute bilateral microinjection of midazolam, phenobarbital, and trimethadione into the SNr protected rats against focal onset seizures induced by ip-injected pilocarpine, while phenytoin was ineffective, and ethosuximide was proconvulsant [212].

Many studies showed that the seizure-controlling properties of the SNr depend on subregion-, age-, and sex-specific features, and on rat strain [213,214,215]. In our review, we only consider studies in adult animals, and most of the studies cited have been performed in male animals. Local manipulations of the SNr can be anticonvulsant, proconvulsant, or without effect depending on the nigral subregion, age, and sex of the animals, but probably can also depend on seizure type investigated [216]. For example, cytoarchitecture, GABA_A_ receptor subunit composition, glutamate receptor distribution, and anatomical connections differ between anterior and posterior SNr (aSNr, pSNr), with higher GABA immunoreactivity levels in the aSNr compared to pSNr in both sexes, but with higher levels in female compared to male rats [215,217]. Kindling-induced alterations in electrophysiological features also differ between the two nigral subregions as investigated in female rats [177]. Muscimol microinjected into the aSNr of amygdala-kindled rats elevated electrographic seizure thresholds in males [200], but not in female rats [77], emphasizing the need for a thorough definition of factors influencing the outcome of SNr modulation. This also includes the choice of the drug and seizure model because bilateral microinjection of vigabatrin into the SNr was anticonvulsant in the female amygdala-kindled rat [198], although nigral subregions have not been differentiated in this early study. Bilateral microinjections of vigabatrin into aSNr as well as pSNr elevated clonic seizure threshold in the ivPTZ-ST test in female rats [204]. Subregional differences concerning seizure-outlasting electrophysiological alterations as well as the outcome of targeted drug delivery have also been described for other basal ganglia regions, such as the striatum in rats (e.g., [176,218]. Further drug delivery studies targeting basal ganglia should take these factors into account. In addition, most studies showed that bilateral modulation of basal ganglia targets is necessary to induce pronounced antiseizure effects. Typically, unilateral manipulations were either without antiseizure effect or the effects were less pronounced. This requirement suggests that seizure suppressive actions evoked from SNr and STN involve mainly lateralized output pathways.

These and other reports initiated further acute intracerebral drug delivery studies investigating putative anatomical basal ganglia sites of anticonvulsant actions. Indeed, all investigated basal ganglia neurons are able to modulate different seizure types depending on the drug delivered. In line with the basal ganglia direct inhibitory pathway (Figure 1), acute bilateral intrastriatal microinjection of NMDA in rats protected against pilocarpine-induced seizures [218] and amygdala-kindled seizures [219]. The anticonvulsant action of NMDA in the striatum was reversed by blocking GABA-mediated inhibition in the SNr, or the entopeduncular nucleus [218]. Likewise, bilateral intrastriatal bicuculline exerted anticonvulsant effects against different seizure types, including amygdala-kindled, pilocarpine-, or kainic acid-induced seizures [220,221].

Apart from the modulation of GABAergic and glutamatergic neurotransmission, modulation of dopaminergic transmission mainly within the striatum has been studied with regard to its ability to modulate seizures. Acute microinjections of dopamine D2 receptor agonists into the dorsal striatum have been shown to control different experimental seizure types, including pilocarpine-induced limbic motor seizures in rats, whereas injections of D2 antagonists in the same region caused proconvulsant effects [222,223]. Noteworthy, acute microinjections of a D2 agonist into the NAcc of fully amygdala-kindled rats protected against focal and secondarily generalized kindled seizures even after unilateral (ipsilateral to the kindling site) drug delivery [224]. Contralateral microinjections were not anticonvulsant. Among other circuitry effects discussed, D2 receptors in the striatum mainly modulate the indirect pathway to the SNr by involving GPe and STN [170].

Acute bilateral microinjection of the GABA uptake inhibitor tiagabine into the GPe of rats exerted anticonvulsant effects on tonic seizures induced by PTZ [225]. However, the benzodiazepine receptor agonist zolpidem was ineffective. In contrast, bilateral injection of the GABA_B_ receptor agonist baclofen completely suppressed the seizures, and this effect was largely abolished by co-injection of a GABA_B_ receptor antagonist, suggesting that in this basal ganglia region, GABA_B_ receptors are more important at least for modulation of tonic seizures [225].

A remarkable nonselective seizure modulation can also be achieved through the STN, which is composed of glutamatergic projection neurons. The STN is part of the indirect basal ganglia pathway and receives strong GABAergic input from the GPe. In addition, the STN receives fast cortical glutamatergic input via the hyperdirect pathway (Figure 1). It monosynaptically excites downstream SNr neurons and modulates nigral discharge patterns. Accordingly, bilateral injection of muscimol into the STN decreased the activity of downstream SNr neurons [226]. The STN also excites GPi neurons, which then could influence limbic seizure circuits through the lateral habenula. The STN is not only highly interconnected within the basal ganglia (and cortical) circuitry. Additionally, it modulates the activity of numerous further brain regions via reciprocal connections with the PPN and further structures outside the basal ganglia [227,228]. Likely because of its widespread connectivity and its strategically prominent role within the basal ganglia, the STN is also relatively nonselective as to the seizure types it can influence.

Acute microinjection of muscimol bilaterally into the STN of rats suppressed absence seizures [229], prevented generalization of amygdala-kindled seizures [201], raised flurothyl-induced clonic seizure thresholds [230], and raised myoclonic and clonic seizure thresholds in the ivPTZ-ST model [57]. The study by [230] also indicated anticonvulsant effects of unilateral muscimol injections into the STN. However, the drug was injected into both hemispheres, but with correct localization of cannula within the STN in only one hemisphere. Bilateral, but not unilateral, acute microinjection of muscimol into STN protected against limbic motor seizures evoked either by iv-injected bicuculline or by application of bicuculline into the anterior piriform cortex of rats [231]. The study further revealed that muscimol injected bilaterally into the STN evoked a mild stereotyped hyperactive behavior, while unilateral intrasubthalamic muscimol injection induced a contralaterally directed postural asymmetry. In contrast, intrasubthalamic delivery of vigabatrin was better tolerated [204]. These drug-dependent differences could be due to the continuous and nonphysiological stimulation of GABA_A_ receptors by muscimol, while vigabatrin probably acts more physiologically depending on the activity of GABAergic connections. Apart from drug-dependent differences in inducing adverse effects upon targeted inhibition of basal ganglia, site-specific effects are known. Muscimol injected into the SNr of rats provoked locomotor activation in addition to the contralaterally directed postural asymmetry observed from STN injection [231]. We recently directly showed that targeting the STN is associated with fewer adverse effects compared to delivery of the same drug (vigabatrin) into the SNr of rats [56]. Furthermore, choreiform dyskinetic movements were observed when muscimol was injected into the SNr, but not into the STN of nonhuman primates [232].

Nevertheless, most animal studies investigating seizure modulation through the basal ganglia traditionally concentrated on the SNr, although it was not clear, which structure (SNr or STN) is more promising regarding seizure control. We were the first who provided a direct comparison between systemic administration of vigabatrin and its targeted acute microinjection into different brain regions, including aSNr, pSNr, and the STN, regarding anticonvulsant efficacy and occurrence of adverse effects [204]. Targeting the STN was the most promising strategy. Bilateral delivery of 10 µg vigabatrin into the STN was more effective in increasing seizure thresholds in the ivPTZ-ST model in rats than either intranigral (10 µg bilateral) or systemic high dose (600 mg/kg ip) administration of vigabatrin. Unilateral vigabatrin delivery into the STN (and drug delivery outside the STN in the other hemisphere) was also effective but to a lesser extent. Furthermore, targeted inhibition of the STN was not associated with the severe adverse effects observed with systemic treatment, i.e., marked sedation and ataxia [204].

Although not directly compared, a cell transplantation study also indicated that bilateral grafting of an inhibitory cell line into the STN might be more effective in raising seizure thresholds than placing the same cell line bilaterally into the SNr [233].

#### 5.1.3. Chronic Basal Ganglia Drug Delivery in Animal Models of Seizures/Epilepsies

Again, most studies were designed as acute injections, while few studies investigated chronic drug delivery into basal ganglia regions in epilepsy models. The first study investigating long-term drug delivery into the basal ganglia in a model of TLE was performed by Kokaia et al. [151]. GABA-releasing polymer matrices implanted bilaterally into the SNr of amygdala-kindled rats blocked generalized (but not focal) seizures for several days [151].

**Figure 4:**
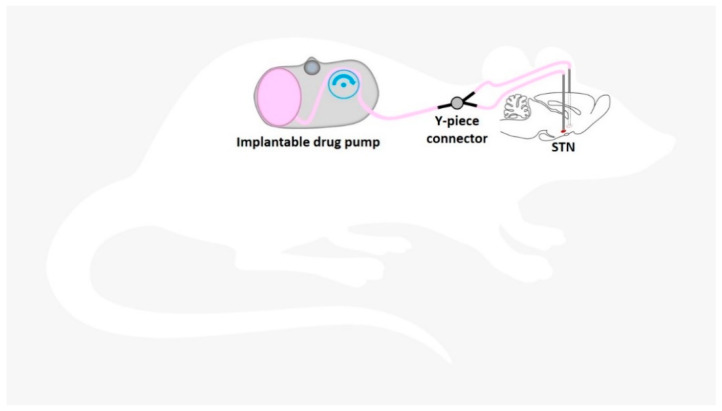
Schematic drawing of the experimental setup used by our group for chronic intracerebral delivery of vigabatrin [56] and muscimol [57] by convection-enhanced delivery in rats. A battery-powered programmable microinfusion pump was implanted subcutaneously and connected to a catheter/cannula system bilaterally targeting the subthalamic nucleus (STN).

The promising results obtained from acute intrasubthalamic vigabatrin delivery [204] described above prompted us to investigate chronic CED of vigabatrin into the STN or SNr [56] and muscimol into the STN [57] of rats. In both studies, drugs were delivered continuously by means of an implantable microinfusion pump connected to intracranial cannulas (Figure 4). Bilateral infusion of 10 μg/day vigabatrin over 3 weeks into the STN almost completely inhibited the GABA-degrading enzyme GABA transaminase (GABA-T) and strongly increased GABA levels in the target region [56]. This was associated with a significant elevation in myoclonic and clonic seizure thresholds, determined once weekly by iv infusion of PTZ (ivPTZ-ST model). In part of the rats, tolerance to the antiseizure effect developed upon continuous drug delivery. There were no indications of vigabatrin-induced lesions. Lower doses were less effective. Bilateral infusion into the aSNr was less effective and induced more severe adverse effects (intense circling in most rats and hypolocomotion and sedation) than intra-STN infusion at the highest dose tested (mainly moderate hypolocomotion and sedation). Unilateral intrasubthalamic infusion was also less effective, both in the ivPTZ-ST model as well as in the amygdala-kindling model [56]. Preliminary data also indicate dose-dependent anticonvulsant effects by CED of muscimol over three weeks into the STN in the ivPTZ-ST model in rats [57]. Again, tolerance occurred in some of the rats during continuous drug delivery. We currently investigate if discontinuous (intermittent) drug delivery will prevent the development of tolerance, similar to the observation after discontinuous transmeningeal muscimol delivery in rats [65].

The exact mechanisms underlying the antiseizure effects of basal ganglia manipulations are not fully understood. Early activation of the cortex and SNr emphasizes the important role of these structures in generalized amygdala-kindled seizures [234]. It is assumed from numerous studies that among other mechanisms, GABAergic efferents from the SNr (and GPi) provide inhibition of downstream anticonvulsant zones. Direct inhibition of the SNr or indirect inhibition via reduction of the STN excitatory drive on SNr neurons could disinhibit those anticonvulsant zones within the dorsal midbrain, the PPN, and/or thalamic regions [38,41,167,192]. In addition, reciprocal connections between basal ganglia and limbic circuitry are thought to be involved in the suppression of limbic seizures (Figure 1). The STN, with its widespread connectivity with other basal ganglia as well as cortical and limbic structures, is suggested to utilize multiple pathways in addition to simply modulating nigral activity during seizure suppression [39]. This is supported by the stronger anticonvulsant effects induced by intrasubthalamic compared to intranigral vigabatrin delivery [204].

#### 5.1.4. Lack of Clinical Trials of Drug Delivery into Basal Ganglia in Humans with DRE

In summary, the basal ganglia, especially the SNr and the STN, were profoundly investigated as a common seizure gating and control mechanism, being relatively nonselective as to the type of seizure or seizure origin they can influence, which is a highly attractive feature with regard to translation into clinical application. Clinical studies support the concept of an inhibitory role of the basal ganglia during TLE and other types of epileptic seizures [36,168]. Most obvious in this respect, high-frequency deep brain stimulation (DBS) targeting the STN is a clinically used treatment approach, which repeatedly proved anticonvulsant efficacy in some of the sysDRE patients [235,236]. Nevertheless, intraparenchymal drug delivery targeting basal ganglia has not yet been investigated clinically in patients with sysDRE. However, Heiss et al. [164] determined the safety and behavioral effects of CED of muscimol injected bilaterally into the STN of nonhuman primate rhesus macaques and revealed dose-related hyperkinetic movements that resolved after stopping the infusion. Muscimol was not toxic to primate brain tissue [164]. Experience with intrasubthalamic drug delivery in humans comes from a clinical trial in patients suffering from Parkinson’s disease. Lidocaine and muscimol were injected over several minutes into the STN of six human patients with Parkinson’s disease, resulting in improved motor symptoms [237].

### 5.2. Thalamic Regions

The thalamus is a complex structure containing multiple nuclei with diverse functional roles, including relaying information between limbic structures, basal ganglia, cortical regions, and brain stem areas [170,238]. In a simplified view, the motor portions of the GPi and SNr project to specific motor-related areas of the thalamus, including the ventral lateral nucleus (VL), ventral anterior nucleus (VA), and centromedian nucleus (CM) [170], while limbic structures such as the amygdala and limbic cortex are strongly interconnected with numerous thalamic regions including the anterior nucleus (AN), mediodorsal thalamus (MD), and also CM [238]. The feasibility of seizure control by targeting different thalamic nuclei, such as the AN mainly for TLE patients and the CM mainly for generalized epilepsies, is well known from clinical use of DBS [36,236]. Although mediated by different mechanisms, acute microinjection of muscimol into the AN in rats delayed the occurrence of pilocarpine-induced generalized seizures, while bicuculline injections into the AN were proconvulsant [239]. However, muscimol induced hypotonia at effective doses [239]. Bilateral muscimol and vigabatrin microinjections into the AN of guinea pigs terminated seizure discharges in PTZ-induced generalized seizures [240].

The DM, which is strongly connected to the amygdala, piriform cortex, and other limbic structures, is known to have an important role in the propagation and modulation of focal to bilateral tonic-clonic seizures [38,143,241,242]. Acute microinjection studies targeting the DM in rats showed that drugs, which enhance excitatory drive or block GABA prolonged kindled seizure duration, while enhanced GABA activity by muscimol reduced seizure duration [243]. Likewise, bilateral microinjections of the NMDA receptor antagonist APH or of muscimol into the MD prior to ip injection of pilocarpine protected against limbic seizures in rats [244]. Ipsilateral microinjection of muscimol, vigabatrin, and an antagonist of the α-amino-3-hydroxy-5-methyl-4-isoxazolepropionic acid (AMPA) subtype of glutamate receptors, but not APH as an antagonist against the NMDA receptor into the MD inhibited generalization of limbic seizures induced by bicuculline injections into the anterior piriform cortex in rats [241]. To fulfill the need that clinical applicability requires prolonged anticonvulsant effects, chronic drug delivery into the DM was investigated. Continuous bilateral GABA infusion into the DM by means of osmotic minipumps connected to a catheter system raised generalized, but not focal, seizure thresholds during one week of infusion in amygdala-kindled rats [61]. No neurologic adverse effects were observed in rats.

Further thalamic regions were investigated for their ability to modulate limbic seizures, for example, the ventromedial (VM) and the posterior nucleus (PO) of the thalamus [245]. Muscimol, vigabatrin, and baclofen suppressed flurothyl-induced seizures in rats when microinjected bilaterally into the PO, but not when injected into the VM [245].

### 5.3. Further Regions

Considering the ample evidence available on the role of basal ganglia in limbic seizure control, it was obvious to investigate basal ganglia target structures apart from thalamic regions for their role in pharmacological seizure modulation. Especially the SC (nigrotectal pathway), but also the PPN (nigrotegmental pathway), were investigated by intracerebral drug delivery approaches to define their role and verify their efficacy in limbic seizure control. The SC is reciprocally connected with basal ganglia regions and sends efferents to thalamic and brain stem regions [173].

As mentioned in chapter 5.1, the SNr provides tonic inhibitory influence onto the deep/intermediate layers of the SC and inhibition of these GABAergic nigral neurons is well-known to suppress numerous seizure types with different origins. Accordingly, disinhibition of the deep layers of the SC exerts an anticonvulsant action, as was proved by the observation that bilateral, but not unilateral, application of bicuculline to the SC attenuates limbic motor seizures induced by unilateral bicuculline injection into the anterior piriform cortex in rats [174]. The mechanism by which disinhibition of the deep/intermediate layers of the SC exerts anticonvulsant actions are not yet understood, although a contribution of connections with lower brain stem regions including areas in pons and medulla has been suggested (e.g., [246,247].

The importance of the SC was confirmed and extended by recent optogenetic studies in rats, showing that activation of deep/intermediate layers of the SC neurons suppressed seizures originating in diverse brain networks, including the limbic system [172]. Unilateral optogenetic stimulation also suppressed seizures but was accompanied by behavioral adverse effects such as contraversive posturing, freezing, or startle response in some of the rats. Optogenetic inhibition of SNr cells suppressed generalized seizures evoked by PTZ, focal bicuculline-induced seizures evoked from the anterior piriform cortex, absence seizures evoked by gamma-butyrolactone, and audiogenic seizures in genetically epilepsy-prone rats [248]. Noteworthy, these effects were reproduced by silencing nigrotectal projections, while silencing nigrotegmental terminals reduced only absence seizures and exacerbated seizures evoked by PTZ, showing that specific efferent projection pathways differentially control different seizure types [248]. In contrast, NMDA and kainate receptor antagonists microinjected bilaterally into the PPN suppressed limbic seizures induced by ip administration of pilocarpine [249].

Scattered data are also available for targeting further subcortical structures, including lateral habenula, which is considered an important relay between basal ganglia and limbic structures such as the hippocampus [175]. For example, NMDA and kainate receptor antagonists and muscimol microinjected bilaterally into the lateral habenula suppressed limbic seizures induced by ip administration of pilocarpine [244,249].

Bilateral microinjections of bicuculline into the zona incerta exerted an anticonvulsant effect on pilocarpine-induced seizures, while muscimol was proconvulsant [250].

## 6. Challenges

Although bypassing the BBB by intracranial drug delivery is a highly attractive approach associated with a number of advantages over systemic drug administrations (see Introduction Section), several challenges must be addressed before translation into clinical use is realistic.

Apart from defining the most promising target site for each subset of sysDRE patients, choosing a drug feasible for long-term intracranial delivery is inevitable. For clinical relevance, several compound requirements should be met, including stability at body temperature, efficacy at concentrations not significantly exceeding physiological osmolarity in brain extracellular compartment, efficacy at physiological pH, water solubility, and lack of local neurotoxicity at therapeutic doses [56,108]. Some further challenges are discussed in the following.

### 6.1. Drug Resistance

Pharmacoresistant epilepsy is a multifactorial and variable phenomenon, for which numerous further mechanisms are under investigation apart from the BBB alterations and peripheral pharmacokinetic mechanisms discussed in the introduction. Some of these further mechanisms will probably also interfere with intracranial drug delivery. According to the target hypothesis [251], epilepsy-induced drug target site alterations at the molecular level cause reduced drug efficacy. However, most drug-resistant patients are resistant to several ASDs acting via different therapeutic targets, thereby supporting resistance mechanisms nonspecific to individual ASDs [20,26].

Other hypotheses include disease etiology, the intrinsic severity model, genetic and epigenetic factors, inflammatory processes, and neural network alterations (for review, see [7,20]. According to the neural network hypothesis, reduced ASD efficacy can be caused by an abnormal (epileptic) neural network formed by epilepsy-induced structural alterations such as neurodegeneration, axonal sprouting, neurogenesis, gliosis, and synaptic reorganization [252]. This may be relevant with regard to target site identification for intracranial drug delivery. Network alterations associated with reduced ASD efficacy are not restricted to the seizure focus (e.g., hippocampus) and related brain areas of the limbic system [29] but are also shown experimentally for more remote structures such as the basal ganglia, e.g., [177,178].

Further aspects may be relevant not only for systemic but also for intracranial drug administration. For example, the concept of altered expression and functionality of drug-metabolizing enzymes in the periphery may also apply for centrally (brain parenchyma and BBB endothelial cells) expressed cytochrome P450 metabolic enzymes [253], thereby maybe adding to ASD resistance even after targeted drug delivery into the brain parenchyma.

Taken together, although peripheral mechanisms for ASD resistance can be circumvented, it is nevertheless likely that intracranial drug delivery induces individually distinct responses definable as responders and nonresponders. Animal experiments performed by our group support this concept by revealing responders and nonresponders after intracranial microinfusions of vigabatrin [56] and muscimol [57], both applied in doses causing tolerable adverse effects. However, these experiments were done in an acute seizure model, meaning that chronic epilepsy-induced resistance mechanisms have not been relevant in these experiments. Epilepsy-independent mechanisms such as genetic and epigenetic factors [254,255] are probably more relevant here, i.e., the genetic and epigenetic hypothesis of ASD resistance is supported by our observation. Obviously, targeted intracranial drug delivery will not be eligible for all DRE patients, and a careful patient selection will be a future challenge for this invasive approach.

As mentioned in chapter 3, numerous animal models not only exist for seizure/epilepsy but also for DRE. Most of them are laborious and therefore not suitable for fast screening. Nevertheless, future research directions should involve investigations of intracranial drug delivery approaches in well-defined animal models of DRE.

### 6.2. Pharmacological Tolerance upon Intracranial Drug Delivery

Similar to systemic ASD administrations [256], the development of tolerance to the antiseizure activity of a drug may lead to a delayed ASD resistance after chronic intracranial drug delivery. Indeed, tolerance developed in response to repeated icv administration of midazolam and allopregnanolone in mice [89] and intraparenchymal infusion of muscimol [57] and vigabatrin [56] in rats, even at low intracranial doses. Tolerance to the antiseizure effect of vigabatrin in the third week of continuous infusion into the STN of rats [56] may be due to feedback inhibition of the GABA-synthesizing enzyme glutamic acid decarboxylase (GAD) in response to markedly elevated GABA levels or due to desensitization of GABA_A_ receptors. However, GAD activity was reduced in the STN of vigabatrin responders as well as nonresponders with no intergroup differences, although low sample sizes limit the validity of this finding [56]. Likewise, GABA-T inhibition and GABA increase were not different between responders and non-responders [56]. Discontinuous intracranial drug delivery (e.g., by programmable microinfusion pumps) or by on-demand drug delivery using closed-loop systems may prevent the development of tolerance [47,65].

### 6.3. Differences in Brain Size and Connectivity between Laboratory Animals and Humans

Most preclinical studies on intracranial drug delivery in seizure/epilepsy models were conducted in rodents. Because of the much smaller volume of brain structures in rodents compared to humans, it is much more difficult to restrict drug distribution to the boundaries of the target region. This entails challenges for safety investigations in rodent models because few mm drug distribution distances in rats or mice may cause neurotoxic adverse effects by infiltrating brain areas surrounding the intended target zone. For example, the STN has a volume of about 0.8 mm^3^ in rats and about 240 mm^3^ in humans [257]. Acute intraparenchymal microinjection [258], as well as chronic CED of vigabatrin [56] into STN or SNr, caused elevated GABA levels in a radius of up to 3–5 mm from the injection site in rats, although a significant contribution of non-target GABA elevations in observed anticonvulsant effects was unlikely [56]. Because of the much larger volume of the STN in humans, it should be easier to confine the drug delivery even by CED to restricted target areas, thereby reducing the risk of adverse effects that may be caused by non-target structures.

Considering the size of human brains, the limited diffusion range rather could be a problem depending on the technical approach used. For example, limited penetration distances (mm range) were described by passive diffusion from polymer implants [49]. However, larger distribution radii can be reached with prolonged drug delivery into the brain using CED [49,166]. Heiss et al. [166] selected rhesus monkeys for their investigation of muscimol distribution in response to prolonged intrahippocampal delivery, because their brains are relatively large and provide a greater volume for drug distribution within the hippocampus and temporal lobe than a rodent brain. However, the infusion rate could not be scaled to reflect the smaller volume of the rodent brain because slower infusion rates increase the amount of muscimol distributed by diffusion, not convection [166].

Apart from size differences, discrepancies in connectivity between rodent and primate brains could influence the outcome of intracranial drug delivery approaches. For example, the STN differs between rats and humans concerning connectivity with other brain areas [227]. Beyond the STN, many further connections were shown to differ between rodents and humans, for example, the degree of segregation in direct and indirect basal ganglia pathways and the connectivity of the NAcc (overview by [36]).

## 7. Conclusions

There is broad experimental evidence that the modulation of part of the epileptic network by targeted intracranial drug delivery is sufficient to control focal seizure initiation, propagation, or generalization. Promising target regions include the seizure focus itself and seizure-controlling structures of the epileptic network remote to the epileptogenic zone. Among the remote regions, especially basal ganglia structures such as the STN were defined preclinically as key nodes exerting a widespread influence on seizure circuitry when pharmacologically modulated. These remote key structures are known to be relatively nonselective as to the type of seizure or seizure origin they can influence. Therefore, they are highly attractive targets for the development of therapeutic intracranial drug delivery approaches for sysDRE patients not eligible for focus resection. However, while most preclinical studies so far utilized acute intracranial bolus injections, long-term targeted delivery is required for suppression of spontaneous recurrent seizures. Future preclinical studies should take this requirement into account. In addition, even though remarkable antiseizure effects can be obtained preclinically by intracranial drug delivery approaches, there are challenges to be solved before this strategy is clinically applicable. These challenges include the risk of development of pharmacological tolerance in response to intracranial drug delivery and pharmacoresistance mechanisms apart from epilepsy-induced BBB alterations.

Although invasive surgical procedures are inevitable, bypassing the BBB by targeted intracranial drug delivery is a promising strategy worth of further development to circumvent BBB-associated drug resistance mechanisms and to lower the risk of systemic and neurologic adverse effects. Until now, very few clinical trials confirming the feasibility of targeted intracranial drug delivery in medically intractable epilepsy patients have been performed. Overall, the existing data show that targeted modulation of neuronal activity is a reasonable research strategy for the investigation and treatment of epilepsy patients resistant to systemic pharmacotherapy.
